# Kombination aus Rhinoshave und fraktional‐ablativer CO_2_‐Lasertherapie zur Feinkonturierung bei ausgeprägten Rhinophymen – eine monozentrische retrospektive Studie mit Langzeitnachbeobachtung

**DOI:** 10.1111/ddg.15692_g

**Published:** 2025-07-14

**Authors:** Conrad Hempel, Anna‐Theresa Seitz, Viktor Schnabel, Till Mittank‐Weidner, Jan‐Christoph Simon, Sonja Grunewald

**Affiliations:** ^1^ Klinik und Poliklinik für Dermatologie Venerologie und Allergologie Universitätsklinikum Leipzig

**Keywords:** CO₂‐Laserbehandlung, Hautchirurgie, Rhinophym, Shaveexzision, CO_2_ laser treatment, Rhinophyma, shave excision, skin surgery

## Abstract

**Hintergrund:**

Zum gegenwärtigen Zeitpunkt gibt es keine effektiven medikamentösen Therapieoptionen, die zur Rückbildung von Rhinophymen führen. Eine Vielzahl an chirurgischen Therapieverfahren ist publiziert, jedoch liegen wenige Daten zur Effektivität vor. Zudem führen diese Therapieoptionen bei übermäßiger Abtragung oder zu großer Hitzeentwicklung häufig zu einer persistierenden narbigen Hypopigmentierung.

**Patienten und Methodik:**

Um eine zu tiefe Abtragung zu vermeiden und die Nasenform genau konturieren zu können wurde eine Kombinationstherapie aus klassischem Rhinoshave zur groben Abtragung mit anschließender Laser‐Feinkonturierung entwickelt. Dabei wird die Ablation fraktioniert durchgeführt, um die Hitzeentwicklung geringer zu halten. Wir berichten über 45 Patienten mit ausgeprägtem Rhinophym, welche im Zeitraum von 2016 bis 2024 in der Universitäts‐Hautklinik Leipzig mit dieser Methode behandelt wurden. Die mediane Nachbeobachtungszeit betrug 51 Monate (6–96 Monate).

**Ergebnisse:**

Die Mehrheit der Patienten war mit dem postoperativen Ergebnis sehr zufrieden oder zufrieden (n  =  26; 92,3 %). Eine Nachblutung wurde berichtet (2,2 %). Es kam zu keiner Wundinfektion. Die Rezidivrate betrug 17,9 %.

**Schlussfolgerungen:**

Die Kombination aus Rhinoshave und fraktional‐ablativer CO_2_‐Laserbehandlung ist ein praktikables und sicheres Therapieverfahren und sorgt bei geringer Rezidivrate für eine hohe Patientenzufriedenheit.

## EINLEITUNG

Als Rhinophym bezeichnet man die mit diffuser Bindegewebs‐ und Talgdrüsenhyperplasie einhergehende Ausprägung der Rosazea der Nase. Die Pathogenese ist bis heute nicht vollständig geklärt.^1^ Angenommen wird eine multifaktorielle Genese, bestehend aus einer neurovaskulären Dysregulation, einer Störung des angeborenen und erworbenen Immunsystems, einer entzündlichen Reaktion auf kutane Mikroorganismen sowie genetischen Faktoren. Das Rhinophym betrifft dabei insbesondere Männer (Ratio 5 1 bis 30 : 1), im Alter über 40 Jahren mit Hauttyp I oder II.^2^, ^3^, ^4^ Betroffen sind ausschließlich die unteren zwei Drittel der Nase mit Beteiligung der Nasenflügel.^5^ Um den Grad eines Rhinophyms beschreiben zu können, wurde der *Rhinophyma Severity Index* (RHISI) eingeführt, welcher fünf klinische Abstufungen, anhand des Grades der Hautverdickung, dem Vorhandensein von Fissuren und knotigen Proliferationen, vornimmt. Die maximale Punktzahl beträgt sechs Punkte. Ein Extrapunkt kann vergeben werden bei dem Vorhandensein einer ausgeprägten Asymmetrie, multipler Zysten oder größerer Gefäße.^6^

Das Rhinophym wird auf Grund seiner zentralen Lage im Mittelgesicht häufig als erheblich störend empfunden. Neben Nasenatmungsbeeinträchtigungen können auch störende Gerüche Grund für eine operative Therapie sein. Zur objektiven Einschätzung kann der RHISI‐Score bestimmt werden. Werte > 3, also eine beginnende Nasenatmungsbeeinträchtigung beziehungsweise erhebliche kosmetische Beeinträchtigung, bestätigen die Notwendigkeit einer operativen Maßnahme.

Zum gegenwärtigen Zeitpunkt gibt es keine medikamentösen Therapieformen, die zu einer Rückbildung eines ausgeprägten Rhinophyms führen. Zahlreiche physikalisch destruktive Therapieverfahren sowie deren Kombinationen sind beschrieben, welche im Wesentlichen drei verschiedene Verfahren nutzen: die rein mechanische Abtragung, die Radiofrequenzablation oder die Laserabtragung.^7^

Die mechanische Abtragung erfolgt klassischerweise mittels Rhinoshave und Dermabrasio.^8^ Nachteil ist eine nur mäßige Möglichkeit der Feinkonturierung insbesondere an den Nasenflügeln und am Naseneingang sowie die fehlende Blutstillung.

Alternativ kann die Abtragung mittels Kryochirurgie oder der Anwendung von Hitze erfolgen.^9^ Diverse Arten von Skalpellen sind verfügbar, insbesondere solche, welche sich auf 150–200°C erhitzen lassen (Shaw scalpel, C2Dx, USA) oder Ultraschall anwenden (Harmonic ultrasound scalpel, Ethicon Endo‐Surgery, USA), um gleichzeitig eine Blutstillung zu erreichen.^10^, ^11^ Elektrochirurgische Ansätze nutzen hochfrequenten Wechselstrom, um gleichzeitig Gewebe zu schneiden und eine Blutstillung zu erreichen.^12^ Bei den genannten hitzebasierten Verfahren wird jedoch ein nicht unerheblicher und manchmal schwer zu steuerndem thermischem Schaden gesetzt. Diese ist die Hauptursache der häufig entstehenden depigmentierten Narben.

Bei der Laserbehandlung ist insbesondere die Anwendung von fraktionierten oder nicht fraktionierten CO_2_‐Lasern und Erbium:YAG‐Lasern beschrieben.^13^, ^14^, ^15^ Die fehlende Möglichkeit der histologischen Gewebeuntersuchung und ein ebenfalls möglicher thermischer Schaden bei größeren Befunden stellen Nachteile dieser Methode dar.

Seltener werden Hydrochirurgiesysteme (wie der Versajet™, Smith & Nephew, UK) eingesetzt, die neben den hohen Investitionskosten auch Nachteile bei der Feinkonturierung aufweisen.^16^ Ebenfalls ist die Anwendung von kaltem Plasma (wie der J‐Plasma, Apyx Medical Corporation, USA) beschrieben.^17^ Der Einsatz von Röntgenstrahlen ist vor dem Hintergrund einer potenziellen Krebsentstehung, insbesondere bei älteren Menschen mit hellerem Hauttyp heutzutage obsolet.^18^

## OP‐TECHNIK

Nach sorgfältiger Farbmarkierung der Ränder des Rhinophyms werden circa 30 mL einer Tumeszenz‐Lokalanästhesie‐Lösung (bestehend aus Lidocain, Ropivacain, Suprarenin und Ringer‐Lösung) infiltriert, bis eine deutliche Aufhellung und Schwellung der Nase zu sehen ist. Nach der Lokalanästhesie wird überschüssiges Gewebe mit einem Handdermatom oder Skalpell (Klinge Nr. 10) abgetragen, um die Grobkonturierung der Nase zu erreichen. Die tieferen Hautschichten werden dabei sorgfältig erhalten, um Narbenbildung zu reduzieren und die Reepithelialisierung zu erleichtern.

**ABBILDUNG 1:**
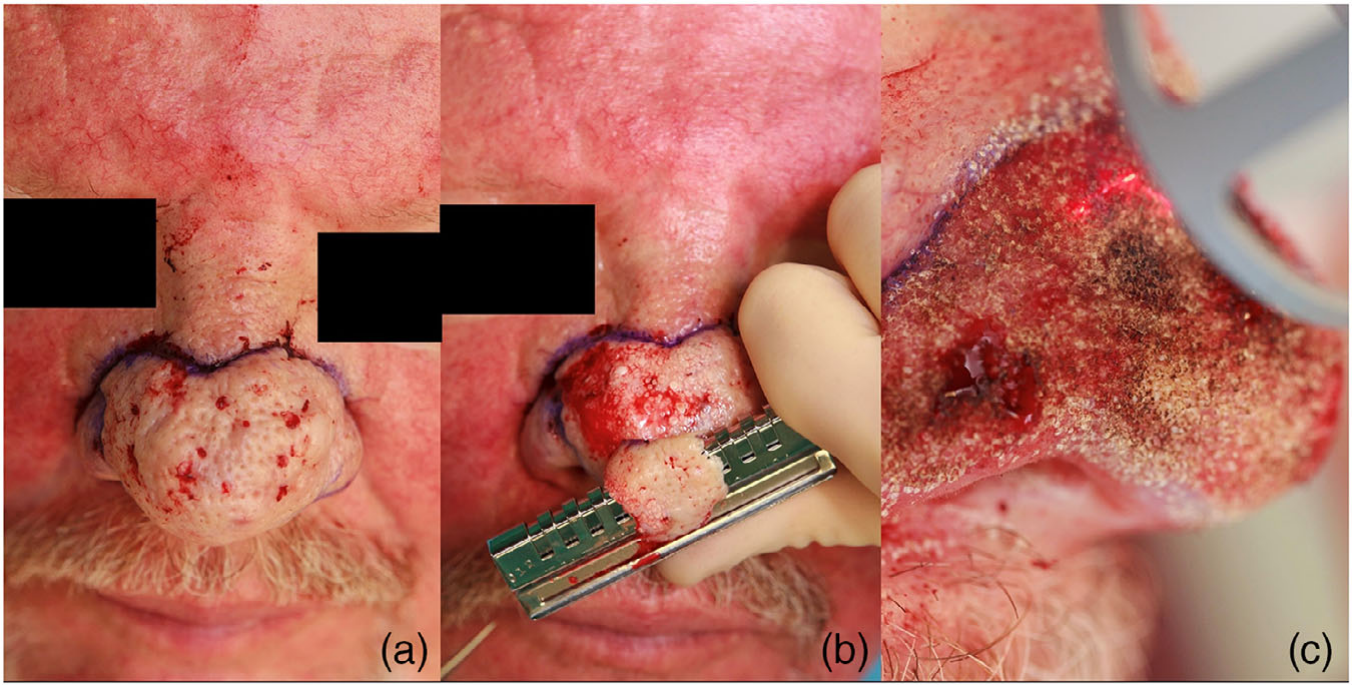
(a) Markiertes OP‐Gebiet nach Infiltrationslokalanästhesie. (b) Abtragung mittels Handdermatom. (c) Fraktionelle CO_2_‐Laseranwendung zur Blutstillung und Feinkonturierung. Die Feinkonturierung erfolgte nach anatomischen Gegebenheiten und persönlichen Fotographien vor Erkrankungsbeginn, tiefere Ablationen wurden dabei durch *multiple passes* erreicht.

Direkt im Anschluss erfolgt eine fraktional‐ablative CO_2_‐Lasertherapie zur Feinkonturierung insbesondere der Nasenspitze und der Nasenflügel (PIXEL CO_2_, Alma Laser, 30 W, 1 ms, 5er Density, die Spotgröße wurde dabei variabel zwischen 4 mm und 6 mm gewählt, bei Wiederherstellung des Sulcus alaris auch noch kleiner) (Abbildung 1). Da CO_2_‐Laser unterschiedliche Parameter (Fluence/Leistung) ausgeben und nur sehr bedingt vergleichbar sind, eignen sich grundsätzlich dafür Parameter mit hoher Leistung beziehungsweise Energiedichte und relativ kurzer Pulsdauer, die Dichte der Ablationskanäle sollte hoch sein. Es sind *multiple passes* notwendig: je tiefer die zu erreichende Kontur liegt, desto mehr. Meist reicht dieses Vorgehen bereits zur Blutstillung aus. Die bipolare Koagulation wird nur bei Bedarf an einzelnen Gefäßen punktuell eingesetzt.

Prinzipiell könnte die Lasertherapie auch im *full ablation mode* erfolgen, dies würde zeitlich sogar schneller gehen. Die Folge wäre jedoch eine stärkere Hitzeentwicklung und somit die Gefahr von bleibenden Narben.

Die Wunde wird 1 bis 2 Tage lang mit Paraffinbeschichteten Gaze (Jelonet^TM^, Smith&Nephew, UK) und antibakterieller Creme (Fucidine^®^, Leo Pharma, Dänemark) abgedeckt und heilt danach offen sekundär unter Belassung der Krusten innerhalb von 1–2 Wochen. Das gesamte entfernte Gewebe wird histologisch untersucht, um klinisch okkulte epitheliale Neoplasien nicht zu übersehen. Routinemäßig wird die Anwendung von Sonnenschutzprodukten nach abgeschlossener Wundheilung empfohlen.

## METHODE

Zusätzlich zur retrospektiven Analyse des Patientenkollektivs einschließlich Fotodokumentation führten wir eine standardisierte telefonische Nachbefragung durch, wobei eine mündliche Einwilligung zur Teilnahme an dieser Datenerhebung erteilt wurde. Es wurde nach einer graduellen Einschätzung der Zufriedenheit gefragt (sehr zufrieden, zufrieden, neutral, unzufrieden und sehr unzufrieden). Mittels Ja‐Nein‐Fragen wurden das Auftreten ästhetisch störender Narben, ein Rezidiv mit gegebenenfalls erneuter Notwendigkeit einer operativen Versorgung, das Auftreten von Komplikationen, die Durchführbarkeit der Operation in örtlicher Betäubung, die Weiterempfehlungswahrscheinlichkeit sowie eine Selbsteinschätzung zur Dauer der Wundbehandlung evaluiert. Ein entsprechendes Votum der Ethikkommission der Universität Leipzig zur Datenerhebung und ‐auswertung lag vor (268/24‐ek). Die Operationstechnik blieb im gesamten Zeitraum unverändert, ebenso die verwendete Instrumentierung. Alle Rhinophymeingriffe wurden in diesem Zeitraum von derselben Operateurin durchgeführt.

## ERGEBNISSE

In dieser monozentrischen retrospektiven Erfassung konnten 46 Patienten mit ausgeprägten Rhinophymen (RHISI‐Score > 3) evaluiert werden, die im Zeitraum von August 2016 bis Februar 2024 mit dieser Technik an der Universitäts‐Hautklinik Leipzig operativ versorgt wurden. Es handelt sich dabei aufgrund des Schweregrades um stationäre Fälle. Bei den kurzfristigen Kontrollen wenige Monate nach Therapie zeigten alle Patienten ein sehr zufriedenstellendes Ergebnis.

Bezüglich des längerfristigen postoperativen Ergebnisses erfolgte eine telefonische Befragung. Es konnten 27/45 (60 %) Patienten erreicht werden. Die mediane Nachbeobachtungszeit betrug 51 Monate (6–96 Monate). Ein Patient verstarb in der Nachbeobachtung an einer behandlungsunabhängigen Ursache. Die erhobenen Daten wurden mit SPSS 29 (IBM, USA) analysiert.

**ABBILDUNG 2:**
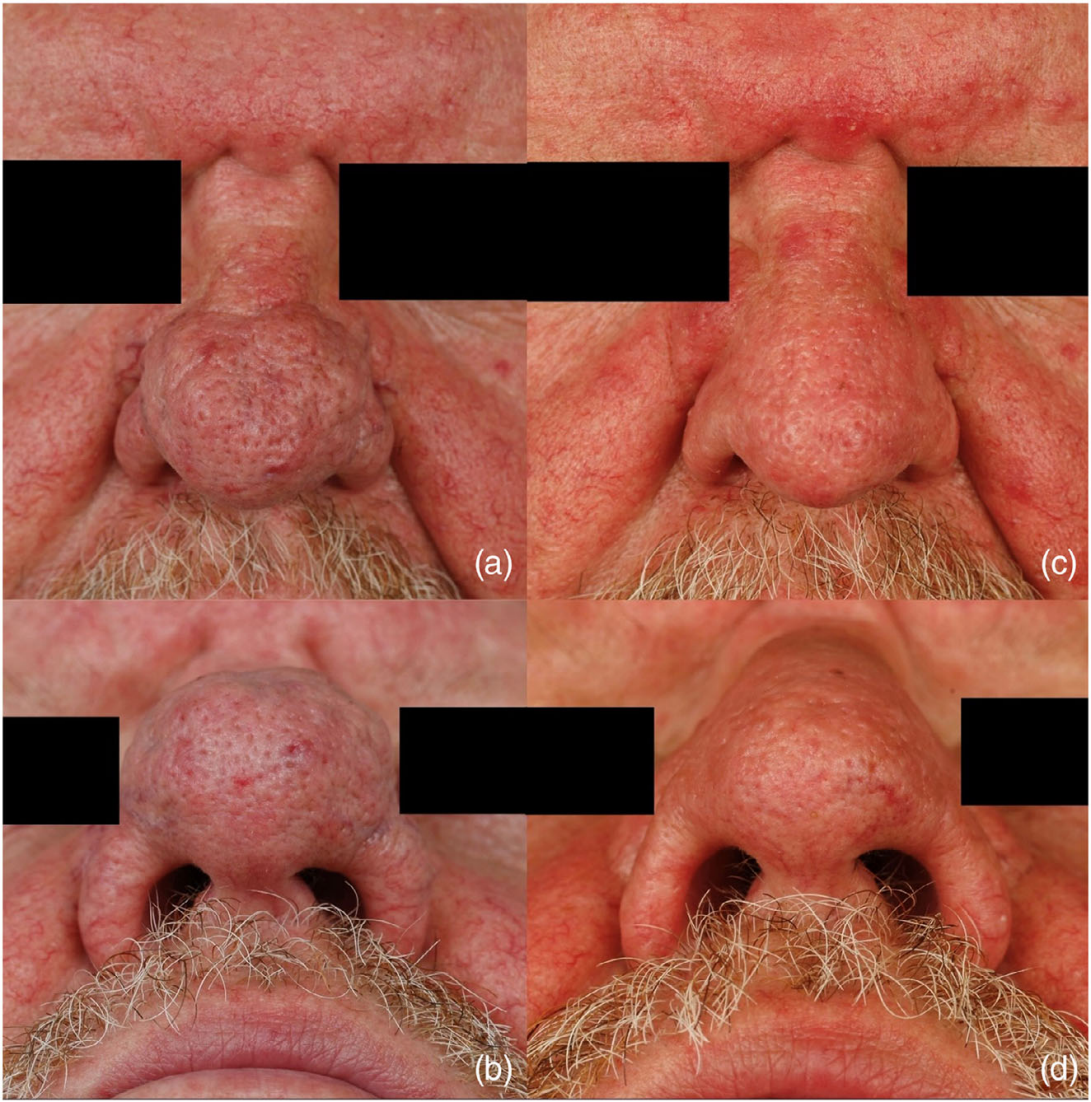
(a) Patientenbeispiel 1: präoperative Aufnahme frontal. (b) Präoperative Aufnahme Naseneingang. (c) Ergebnis 3 Monate postoperativ frontal. (d) Ergebnis 3 Monate postoperativ Naseneingang.

**ABBILDUNG 3:**
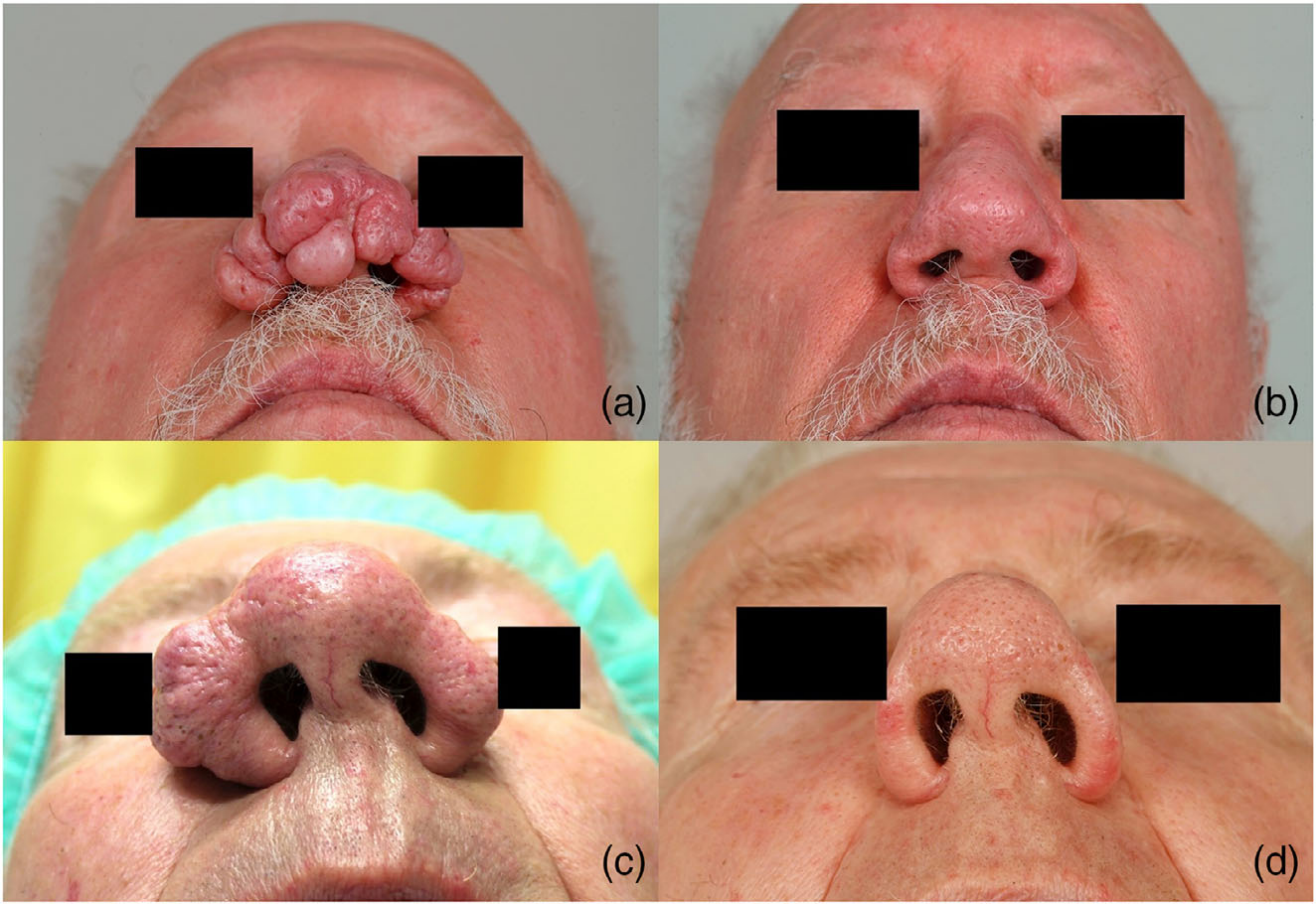
(a) Patientenbeispiel 2: Präoperative Aufnahme. (b) Ergebnis 3 Monate postoperativ. (c) Patientenbeispiel 3: Präoperative Aufnahme. (d) Ergebnis 3 Monate postoperativ.

Der überwiegende Anteil unserer Patienten war männlich (n  =  43; 91,5 %) und litt an einem fortgeschrittenen Rhinophym (durchschnittlicher RHISI‐Score 4,4) (Tabelle 1). Das mediane Alter zum Operationszeitpunkt betrug 67 Jahre (37–81 Jahre). Die Mehrheit der Patienten war langfristig mit dem postoperativen Ergebnis „sehr zufrieden“ oder „zufrieden“ (n  =  25; 92,6 %). Kein Patient erlitt funktionelle Beeinträchtigungen. Zwei Patienten (n  =  2; 7,4 %) zeigten sich „unzufrieden“ mit dem kosmetischen postoperativen Ergebnis, beide hatten dabei ein Rezidiv ihres Rhinophyms. Einer der „unzufriedenen Patienten“ empfand zusätzlich die OP in örtlicher Betäubung als Belastung, der andere berichtete von einer ästhetisch störenden Narbenbildung. Kein Patient zeigte sich „sehr unzufrieden“ (Tabelle 2). Eine moderate Nachblutung trat bei einem Patienten unter dem Thrombozytenaggregationshemmer Clopidogrel auf, welche eine bipolare Blutstillung erforderte. Es kam postoperativ zu keiner Infektion (Tabelle 3). Die deutliche Mehrheit der Patienten empfand die Durchführung der OP in örtlicher Betäubung nicht als Belastung (n  =  24; 88,9 %) und würde das OP‐Verfahren weiterempfehlen (n  = 25; 92,6 %). Lediglich ein Patient empfand die Wundheilung als lang. Die Rezidivrate lag bei 17,9 %. Ein Patient berichtet über eine subjektiv störende Narbenbildung (n  =  1; 3,7 %) ohne Wunsch einer Folgebehandlung (Abbildungen 2, 3).

**TABELLE 1:** Epidemiologische Daten von 46 Patienten mit Rhinophym.

Geschlecht	
Männlich	43 Patienten (95,6 %)
Weiblich	2 Patienten (4,4 %)
Mittlerer RHISI‐Score	4,4
Durchschnittalter zur OP	63,7 Jahre
Medianes Alter zur OP	67 Jahre

**TABELLE 2:** Patientenzufriedenheit.

Zufriedenheitsgrad	n (%)
Sehr zufrieden	19 (70,4 %)
Zufrieden	6 (22,2 %)
Neutral	0 (0 %)
Unzufrieden	2 (7,4 %)Gründe für Unzufriedenheit (Mehrfachauswahl möglich): ‐ Rezidiv 100 % ‐ Narbenbildung 50 % ‐ Belastung durch Lokalanästhesie 50 %
Sehr unzufrieden	0 (0 %)
Ästhetisch störende postoperative Narbenbildung	1 (3,7 %)
Auftreten eines Rezidivs	5 (17,9 %)
Haben sie die Durchführung der OP in örtlicher Betäubung als Belastung empfunden?	Ja: 3 (11,1 %)
	Nein: 24 (88,9 %)
Haben sie die Wundheilung als lang empfunden?	Ja: 1 (3,7 %)
	Nein: 26 (96,3 %)
Würden sie das OP‐Verfahren weiterempfehlen?	Ja: 25 (92,6 %)
	Nein: 2 (7,4 %)

**TABELLE 3:** Postoperative Komplikationen.

Komplikation	n (%)
Nachblutung	1 (3,5 %)
Infektion	0 (0 %)

## DISKUSSION

In der Literatur finden sich sehr wenige publizierte längerfristige postoperative Ergebnisse nach Rhinophymbehandlung. Die Rezidivrate unserer kombinierten Methode ist im Vergleich zur alleinigen Shaveexzision deutlich niedriger (17,9 % vs. 47 %).^6^ Die zusätzliche Laserbehandlung scheint hier einen Mehrwert zu bringen. Möglicherweise kann durch den thermischen Effekt des Lasers, insbesondere in tieferen Hautschichten, eine langfristige Stabilisierung des kollagenen Fasergerüstes erreicht werden, was sich präventiv auf die Ausbildung eines Rhinophyms auswirkt. Angaben zu langfristigen Rezidivraten anderer Methoden, insbesondere von Kaltplasma, fehlen in der Literatur.

Typische Langzeitnebenwirkungen anderer Verfahren der Rhinophymbehandlung, insbesondere Hypopigmentierungen, haben wir nicht feststellen können. Lediglich in einem Fall wurde uns von einer persistierenden Narbenbildung berichtet, hierbei erfolgte die Abtragung beim Rhinoshave versehentlich zu tief. Limitation der vorliegenden Studie ist der retrospektive Charakter und der fehlende direkte Vergleich zu anderen OP‐Methoden.

Im Vergleich zu einer alleinigen CO₂‐Lasertherapie im voll ablativen Modus (Continuous Cutting Mode und Resurfacing Mode) zeigen sich vergleichbare Ergebnisse. In einer Studie, welche die Ergebnisse von 124 Patienten berücksichtigt, findet sich 3 Monate nach der letzten Laserbehandlung, nach Beurteilung durch die Behandler selbst, ein gutes bis sehr gutes Ergebnis in 118/124 Fällen (95,2 %) und lediglich in sechs Fällen (4,8 %) ein schlechtes Ergebnis. Zu berücksichtigen ist dabei jedoch, dass die Behandlung in neun Fällen mehr als einmal durchgeführt wurde (7,3 %). Narbenbildung und Hypopigmentierungen wurden jeweils in vier Fällen (3,2 %) berichtet.^19^ Nachteil der alleinigen Laserbehandlung ist neben der größeren Hitzeentwicklung die fehlende Möglichkeit der histologischen Aufarbeitung und – insbesondere bei größeren exophytischen Rhinophymen – der höhere Zeitaufwand.

Mittels der hier vorgestellten kombinierten Methode können schonend und zeitsparend größere Gewebemengen abgetragen werden und gleichzeitig eine Feinkonturierung entlang der konvexen und konkaven Areale an den Nasenflügeln mittels CO_2_‐Lasertherapie erreicht werden, die mit einer alleinigen Shaveexzision nicht möglich wären.

Die Vielzahl der veröffentlichten Therapieoptionen zeigt, dass es noch keine einzig richtige gibt. Durch die Kombination verschiedener Methoden lassen sich die Vorteile jeder einzelnen Methode nutzen.^20^, ^21^ Der geringe Anteil an weiblichen Patienten in unserer Kohorte ist konsistent mit der Literatur.^7^

Die Tumeszenz‐Lokalanästhesie erleichtert die Durchführung von Shave‐Exzision und Laserbehandlung, wobei die Zugabe von Adrenalin das Blutungsrisiko senkt und die Kombination von Ropivacain und Lidocain für eine langanhaltende Anästhesie sorgt. Dies wurde von unseren Patienten mehrheitlich nicht als Belastung empfunden.

Einige Operateure bevorzugen die Durchführung in Vollnarkose, gegebenenfalls in Kombination mit einer Lokalanästhesie, insbesondere bei sehr weichen Rhinophymnasen, um die tatsächliche Nasenform besser erkennen zu können.^22^

Tatsächlich ist die ursprüngliche Nasenform bei ausgeprägten Rhinophymen auch ohne Tumeszenz‐Lokalanästhesie kaum erkennbar und alte Fotos sind meist hilfreicher. Im Vergleich zu einer Vollnarkose ist zusätzlich die Kosten‐ und Zeitersparnis zu erwähnen, bei minimalem Nebenwirkungsrisiko. Insbesondere bei älteren Patienten stellt die Tumeszenz‐Lokalanästhesie ein sicheres und effektives Verfahren da.^23^ Durch Verwendung eines langwirksamen Lokalanästhetikums reduziert sich der postoperative Gebrauch von Analgetika und das Risiko von Arzneimittelnebenwirkungen sinkt.^23^

Die Kombination von Rhinoshave und fraktional‐ablativer Feinkonturierung ermöglicht bei fortgeschrittenen Rhinophymen eine schonende Abtragung auch größerer Mengen Gewebes. Sowohl die histologische Untersuchung als auch eine gute Blutstillung werden dabei erreicht. Zudem führt die Feinkonturierung zu sehr guten kosmetischen Ergebnissen mit minimiertem Narbenrisiko.

Die hier vorgestellte Methode eignet sich unserer Ansicht nach insbesondere für größere exophytische Rhinophymen mit Beteiligung der Nasenflügel. Zur Validierung dieser Studienergebnisse wären zukünftig prospektive, randomisierte, multizentrische Studien, insbesondere Vergleichsstudien verschiedener Therapieverfahren, hilfreich.

## DANKSAGUNG

Open access Veröffentlichung ermöglicht und organisiert durch Projekt DEAL.

## INTERESSENKONFLIKT

Keiner.
